# The Choice of Search Engine Affects Sequencing Depth and HLA Class I Allele-Specific Peptide Repertoires

**DOI:** 10.1016/j.mcpro.2021.100124

**Published:** 2021-07-23

**Authors:** Robert Parker, Arun Tailor, Xu Peng, Annalisa Nicastri, Johannes Zerweck, Ulf Reimer, Holger Wenschuh, Karsten Schnatbaum, Nicola Ternette

**Affiliations:** 1 Nuffield Department of Medicine, Centre for Cellar and Medical Physiology, University of Oxford, Oxford, UK; 2 JPT Peptide Technologies GmbH, Berlin, Germany

**Keywords:** immunopeptidomics, human leukocyte antigen, HLA, major histocompatibility complex, MHC, peptide sequence annotation, MS search engine, peptide spectrum match, database search, *de novo* sequencing, FDR, false discovery rate, HLA, human leukocyte antigen, LC-MS, liquid chromatography mass spectrometry, MHC, major histocompatibility complex, PSM, peptide-spectrum match

## Abstract

Standardization of immunopeptidomics experiments across laboratories is a pressing issue within the field, and currently a variety of different methods for sample preparation and data analysis tools are applied. Here, we compared different software packages to interrogate immunopeptidomics datasets and found that Peaks reproducibly reports substantially more peptide sequences (~30–70%) compared with Maxquant, Comet, and MS-GF+ at a global false discovery rate (FDR) of <1%. We noted that these differences are driven by search space and spectral ranking. Furthermore, we observed differences in the proportion of peptides binding the human leukocyte antigen (HLA) alleles present in the samples, indicating that sequence-related differences affected the performance of each tested engine. Utilizing data from single HLA allele expressing cell lines, we observed significant differences in amino acid frequency among the peptides reported, with a broadly higher representation of hydrophobic amino acids L, I, P, and V reported by Peaks. We validated these results using data generated with a synthetic library of 2000 HLA-associated peptides from four common HLA alleles with distinct anchor residues. Our investigation highlights that search engines create a bias in peptide sequence depth and peptide amino acid composition, and resulting data should be interpreted with caution.

## Highlights

• Standardized performance evaluation of four search engines in immunopeptidomics.
• Search space and spectral ranking drive differences in sensitivity.
• Observed bias in allele-specific repertoire is driven by the amino acid composition.
• Validation using a community-accessible synthetic HLA-I peptide standard.

## Graphical Abstract

**Figure:**
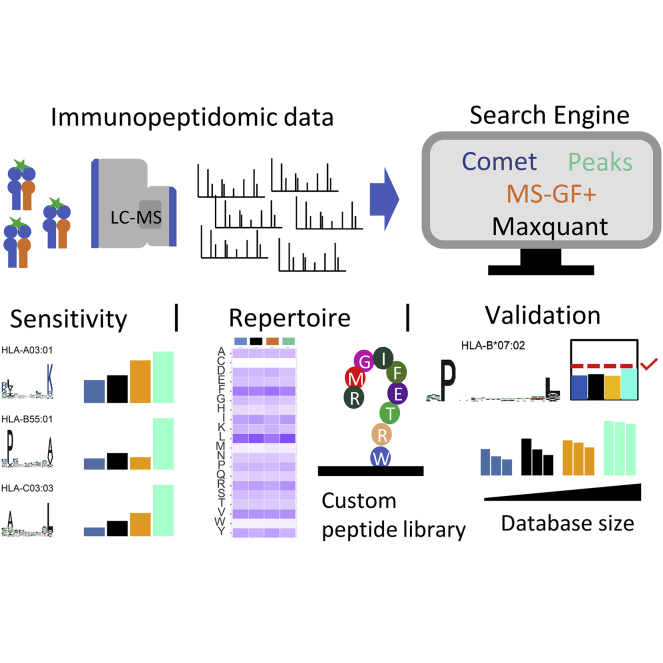

## In Brief

We compared the performance of four search engines for immunopeptidomics data analysis. Differences in sensitivity were associated with the database search space and spectral ranking. In addition, we observed biases in the proportion of peptides identified for individual HLA alleles, which was driven by lower performance for peptides with high hydrophobic amino acid frequency. We validated these findings using a synthetic HLA peptide library and concluded that Peaks is the most sensitive and least biased out of the four tested engines.

The identification of peptide ligands presented by the major histocompatibility complex (MHC; human leukocyte antigen (HLA) in humans) is a vital step in understanding how the cellular immune system recognizes and eliminates infected or malignant cells (1). In humans there are up to six highly polymorphic classical class I HLA proteins expressed. Each allele variant restricts the repertoire of its 8 to 14 mer peptide ligands to distinct amino acid motifs, with anchor residues predominantly at position (P) 2 and 9, and with positions P3 and P5 also being important for some alleles (2).

In recent years, the identification of HLA peptide ligands has been revolutionized by advancements in the sensitivity, speed, and fragmentation efficiency of modern mass spectrometers (3, 4, 5). Alongside the improvements in data acquisition, novel computational algorithms for the identification of HLA peptide sequences have been developed (MS rescue, MHCquant, DeepRescore) but are not yet routinely implemented in most search engines used in immunopeptidomics laboratories (4, 6, 7, 8).

The majority of bioinformatic tools currently used to identify spectra from HLA peptides were originally developed for classical shotgun proteomics. These programs are frequently applied to datasets where trypsin was used to provide a set of peptides restricted to R or K residues at the C-terminus, which are highly suitable for mass spectrometric analysis (9, 10). Such peptides provide a highly confident search space where spectral matches can be made at high sensitivity to the most likely mature and stably expressed proteins found in the cell (11). Immunopeptidomics is clearly distinct from trypsin-based analysis of proteomes, as it requires highly sensitive peptide identification methods in large search spaces that account for the diverse sequence motifs created by polymorphisms in *HLA* *loci* (12).

Several approaches have been developed that involve utilizing multiple search engines or post-hoc rescoring of peptide-spectrum matches (PSM) (7, 13, 14). Results from these studies clearly indicate a need for improving the current search engines and highlight differences in engine performance.

To investigate these observed differences in sequence annotations across search engines, we here present a systematic comparison of four mass spectral peptide identification tools used in immunopeptidomic research. Additionally, we generated and tested a library of 2000 synthetic HLA peptides covering four common HLA alleles. Our results demonstrate that the Peaks database search (Peaks; Bioinformatics solutions) provides significant improvements in sensitivity and a reduction in peptide sequence bias when compared with the classical database search engines Comet, MS-GF+, and Maxquant.

## Experimental Procedures

### Ethical Approval

All human data were downloaded from PRIDE database and derived from published studies that state they were approved by ethics committees and samples were obtained with informed consent.

### Preparation of Antibody-Conjugated Beads

One milliliter Protein A-sepharose beads (GE Healthcare) were washed in 50 mM borate, 50 mM KCl (pH 8.0) solution, and then incubated with 2 mg of antibody slowly rotating 1 h in cold room. Beads were washed with 0.2 M triethanolamine (pH 8.2) and cross-linked with 40 mM dimethyl pimelimidate dihydrochloride (DMP) (Sigma) (pH 8.3) for 1 h at room temperature. Ice-cold 0.2 M Tris buffer (pH 8.0) was added to stop the reaction, and beads were washed with 0.1 M citrate (pH 3.0), and finally 50 mM Tris (pH 8.0).

### MHC Class I Immunoprecipitation

5 × 10^8^ cells were pelleted and lysed in 10 ml lysis buffer (0.5% IGEPAL 630, 150 mM NaCl, 50 mM Tris (pH 8.0) plus protease inhibitor cocktail (Roche)) for 30 min. Lysates were centrifuged at 300*g* for 10 min and then at 15,000*g* for 60 min and incubated with 1 ml antibody-protein G-Sepharose beads (GE) (1 ml) overnight. Beads were washed by 50 mM Tris buffer (pH 8.0) containing first 150 mM NaCl, then 450 mM NaCl, and no salt in the final wash. Complexes were eluted with 5 ml 10% acetic acid and dried.

### High-Performance Liquid Chromatography Peptide Fractionation

MHC complexes were resuspended in 120 μl of loading buffer (0.1% trifluoroacetic acid (TFA), 1% acetonitrile (ACN) in water) and fractioned by RP-HPLC using an Ultimate 3000 high-performance liquid chromatography (HPLC) system (Thermo Scientific) and 4.6 × 50 mm ProSwift RP-1S column (Thermo Scientific) with 10 min gradient from 3% to 30% ACN in 0.1% TFA in water at a flow rate of 1 ml/min. Alternate fractions containing peptides were separated into odd and even samples, dried, and resuspended in 20 μl of loading buffer and analyzed by LC-MS.

### Protein Lysate Preparation and Digestion for Mass Spectrometry Analyses

DoTc2 and HeLa cells were cultured to 80% confluence in Dulbecco’s modified Eagle medium (Sigma) supplemented with 10% heat-inactivated fetal calf serum, 2 mM L-glutamine, and 100U penicillin/ml, and was incubated at 37 °C in 5% CO_2_. Cell pellets were collected by centrifugation and lysed in lysis buffer (0.5% (v/v) IGEPAL 630, 50 mM Tris pH 8.0, 150 mM NaCl, and one tablet cOmplete Protease Inhibitor Cocktail EDTA-free (Roche) per 10 ml buffer) at 4 °C then centrifuged at 3000*g* for 10 min followed by a 20,000*g* spin step for 15 min at 4 °C. Supernatants were measured for protein content (BCA assay, Thermo Fisher) and were purified by chloroform/methanol precipitation. Protein pellets were dissolved in 6 M Urea, 100 mM Tris-HLC pH 7.4, and 5 mM DTT for 30 min. Next, cysteine residues were alkylated with 20 mM iodoacetamide (IA) for 15 min followed by addition of DTT to 20 mM to react with residual IA for 15 min. Lysates were diluted to a final urea concentration of 2 M, and trypsin or elastase was added at a 1:50 enzyme to protein ratio, followed by incubation at 37 °C for 16 h. Sample cleanup was performed with a C18 column (Waters Oasis SPE kit).

### Synthesis of Synthetic Standard

Synthetic peptides were individually synthesized by solid-phase synthesis on cellulose membranes as described previously (15). During synthesis, a carbamidomethylated cysteine building block was used for cysteine to eliminate the need for cysteine modification before MS analysis. Peptides were cleaved from the membrane into pools of 250 peptides each.

### Mass Spectrometric Analysis

Peptide mixtures were dissolved in loading buffer (1% Acetonitrile, 0.1% Trifluoroacetic acid), and 200 fmols/peptide were analyzed by an Ultimate 3000 HPLC system coupled to a high field Q-Exactive (HFX) Orbitrap mass spectrometer (Thermo Scientific). Peptides were trapped by PepMap 100 C18 columns (ThermoFisher Scientific) before reverse phase separation with a 60 min gradient of acetonitrile 2% to 25%, in 1% DMSO, 0.1% Formic acid at a flow rate of 250 nl/min on a 75 μm × 50 cm PepMap RSLC C18 EasySpray column (ThermoFisher Scientific). Data-dependent acquisition involved one full MS1 scan (120,000 resolution, 60 ms accumulation time, AGC 3 × 10^6^) followed by 20 data-dependent MS2 scans (60,000 resolution, 120 ms accumulation time, AGC 5 × 10^5^), with an isolation width of 1.6 m/z and normalized HCD energy of 25%. Three methods were utilized for analysis of the synthetic standard: (A) considered charge states of 2 to 4, (B) considered charge states of 1 to 4 while (C) involved one full scan 300 to 700 followed by 18 MS2 scans for charge states 2 to 4 followed by one full scan 700 to 1400 followed by two MS2 scans for charge states 1. Dynamic exclusion was set for 30 s. For enzymatic digests normalized HCD was increased to 28% and only 2 to 4 charge states were acquired.

### Raw Data Processing

Mass spectrometry raw data files were downloaded from the PRIDE partner repository or MassIVE from the following projects: PXD007635, PXD004894, PXD007635, PXD009531, MSV000080527. Raw data files were converted to mzXML by MSConvert using 32 bit Thermo RAW defaults (v3.0.19014) analyzed in COMET (2019013), Maxquant (v.1.6.1.0), MS-GF+ (v.20181015), and PEAKS 8.5 (Bioinformatic Solutions), inputting a protein sequence fasta file containing 20,606 reviewed human Uniprot entries downloaded on 24/05/2018 appended to the same (DECOY) entries after randomization. No enzyme specificity was set (with exception of the tryptic digest, for which “trypsin” was selected), peptide mass error tolerances were set at 5 or 20 ppm for precursors depending on the dataset and 0.03 Da for MS2 fragments and only peptides of length 7 to 25 were considered, for the analysis of the peptide standard and enzymatic digests, “carbamidomethylated cysteine” were considered as fixed modification. A 1% false discovery rate (FDR) was calculated using a decoy database search approach. PSMs were ranked by score best to worst (PEP, SpecEValue, Evalue, and −10logP Score) for each search engine respectively. FDR was calculated as the cumulative sum of decoys as a fraction of all PSMs as described further in the Results section.

Data analysis and plotting were performed with R or Microsoft excel. NetMHCpan 4.1 (http://www.cbs.dtu.dk/services/) was installed locally and utilized to define allele binding predictions (rank score cut-off 0.5 or 2). Peptide sequences were clustered into distinct motifs using MixMHCp v2.1 (https://mixmhcp.vital-it.ch/#/submission) (16, 17). Sequence logos were generated by the Seq2logo2.0 package in R or by MixMHCp. Venn diagrams and UpsetR plots were created using UpsetR and BioVenn packages in R. Amino acid composition enrichment analysis was done using Composition Profiler (http://www.cprofiler.org/) (18). Analysis of variance (ANOVA) was carried in R. Peptide retention time prediction was done using the SpecL program in R (19).

### Experimental Design and Statistical Rationale

Four diverse immunopeptidome datasets from independent laboratories formed the main part of this work. Firstly, our initial observations were found in analysis of our in-house Ovarian cancer cell line data DoTc2 (high-resolution HCD MS2 spectra, HLA∗A03:01, HLA∗B55:01, HLA∗C03:03). Secondly, to provide an extensive dataset with consistent acquisition parameters, we chose 19 Melanoma tissue samples (87 raw files) from which we explored in detail the MM15 patient dataset (high-resolution HCD MS2 spectra, HLA∗A03:01, HLA∗A68:01, HLA∗B27:05, HLA∗B35:03, HLA∗C02:02, HLA∗C04:01). Thirdly, to observe if effects were consistent between laboratories, sample types and acquisition methods, data from an ovarian cancer tissue sample (low-resolution CID MS2 spectra) (1 raw file) and a Glioblastoma tissue sample (high-resolution HCD MS2 spectra, HLA∗A02:01, HLA∗A32:01, HLA∗B27:05, HLA∗B44:02, HLA∗C05:01, HLA∗C02:02) (two raw files) were obtained from the PRIDE repository. Finally, to assess for biases between HLA alleles, we selected 13 datasets acquired from single allele expressing cell lines from two independent studies (high-resolution HCD MS2 spectra). As controls we additionally investigated proteomic data using a tryptic or elastase digestion of HELA cell (two raw files) lysates (high-resolution HCD MS2 spectra). To validate our results, we analyzed a synthetic standard and this sample using three different mass spectrometry methods (nine raw files) (high-resolution HCD MS2 spectra).

## Results

### Comparison of Four Search Engines for Analysis of Immunopeptidomic Data

Four search engines were assessed for performance in the analysis of immunopeptidomics datasets: (1) **COMET**(v.2019013)—an open-source database search tool used in both proteomic and immunopeptidomic workflows (20); (2) **Maxquant** (Andromeda) (v.1.6.1.0)—used for both proteomic and immunopeptidomic studies (21); (3) **MS-GF+** (v.20181015)—a recently developed search engine that is highly adaptable to the dataset under investigation by deriving scores independent of type of spectra acquired (22); (4) **Peaks** (v.8.5)—a commercial search engine that utilizes *de novo* sequencing to score spectra prior to a sequence database search frequently utilized in a broad range of peptidomic studies (23).

Raw data files for class I immunopeptidomic datasets formed the basis of this work. Three datasets that were acquired on high-resolution HCD-type instruments: (1) the cervical cell line DoTc2 cell line (“DoTc2”; in-house), (2) glioblastoma cancer tissue (“Glio”; PXD007635, (24)), (3) 19 Melanoma tissue samples (“MM”; PXD004894, (25)) and one dataset that was acquired on a hybrid mass spectrometer with low-resolution CID, (4) an ovarian cancer tissue sample (“Ova”; (24)), were selected for this study. Raw files were downloaded and processed by MSconvert (ProteoWizard 3.0.19014) into mzXML format, and all files were subsequently searched using the previously described four search engines. To standardize FDR calculations across each search engine, the same human UNIPROT database (downloaded on 24/05/2018, 20,361 entries) with additional randomized (decoy) sequences appended was adopted for all analyses. We also standardized mass tolerances (5–20 ppm), peptide length restriction (7–25 mers), disabled any additional search space constraints and scoring filters, and included all charge states acquired. Each search engine had a unique method of determining sensitivity cutoffs; for COMET, this could not be disabled. To remove any bias, we removed PSMs to the inbuilt FDR approaches and calculated FDR externally based on PSMs to the decoy sequences appended to the human Swissprot database. We validated this approach by comparing it to in-built FDR calculations for the Dotc2 dataset and found an 83% to 95% (DoTc2) consensus (Fig. S1).

**Fig. 1:**
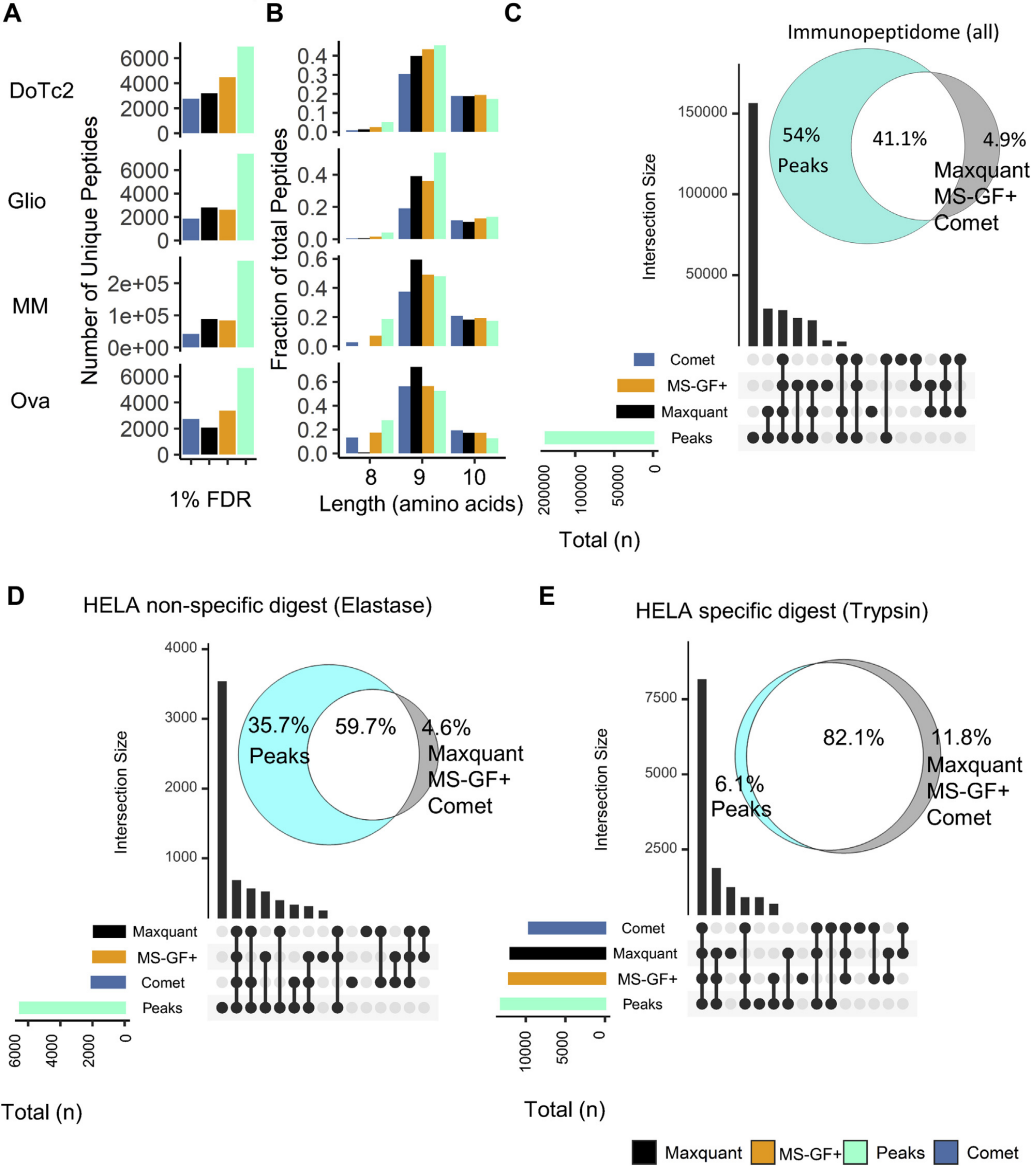
**Comparison of search engine performance at 1% FDR.***A*, number of peptide sequences detected at 1% FDR for each dataset/search engine. *B*, amino acid length distribution for peptides identified by each search engine for each dataset at 1% FDR. *C*–*E*, overlap and unique peptide identifications made by each search engine for the regarding dataset at 1% FDR for (*C*) all immunopeptidomic (*D*) elastase digestion and (*E*) tryptic digestion datasets.

Initially, we assessed the number of peptide identifications reported by each engine using an estimate of global FDR, which was calculated based on the cumulative sum of decoy (B) and target (A) PSMs after peptide ranking by score [SpecEValue (COMET), PEP (Maxquant), Evalue (MS-GF+) and −10logP (Peaks)] for each search engine as follows: [FDR = B/(A + B)]. At an FDR cutoff of 1%, the search engines varied considerably in the number of unique peptides reported for each immunopeptidomic dataset (Fig. 1*A*). The length distributions were as expected for class I immunopeptidomic data; however, there was distinct lack of 8-mer peptides in the Maxquant search results (Fig. 1*B*). The number of identifications reported was consistently higher for Peaks (42%–69%) compared with MS-GF+, Maxquant, and COMET at a 1% FDR cutoff, respectively (Fig. 1*C*). Intersection analysis based on peptide sequences indicated that most peptides (93%–99%) found by COMET, Maxquant, and MS-GF+ were also identified by Peaks. (Fig. 1*C*). Additional sequences identified by Peaks (and other search engines) exhibited similar mass error deviations and predictable retention time characteristics when compared with peptides identified in common (Fig. S2), but, on average, had a lower score (Fig. S3), indicating that the ranking of PSMs is a major factor affecting peptide identification when using FDR in immunopeptidomics (26).

To explore this effect further, we determined the fate of scans identified by Peaks at FDR <1% in the other search engines at a relaxed FDR of 5%. This analysis revealed that between 35% and 86% of the additional peptides identified at 5% FDR were also identified by Peaks at 1% FDR, supporting the hypothesis that the increased performance of Peaks was defined by an improved ranking of peptides in the lower score range (Fig. S4).

To investigate whether these effects were due to the larger search space resulting from unspecific searches, we analyzed data generated by a low-specificity (elastase) enzyme digestion and a specific (trypsin) digest of HeLa cell lysates. The low-specificity digest was able to recapitulate the changes in peptide identification rate observed in immunopeptidomic datasets (Fig. 1*D*). In contrast, high consistency (82%) between the search engines was observed for the specific (tryptic) search space using Peaks for tryptic data as observed previously (Fig. 1*E*) (23).

### Peptide Binding Prediction and Motif Analysis Reveal Search Engine-Specific Differences in the Extent of Reported HLA Allele-Assigned Peptide Repertoires

**Fig. 2:**
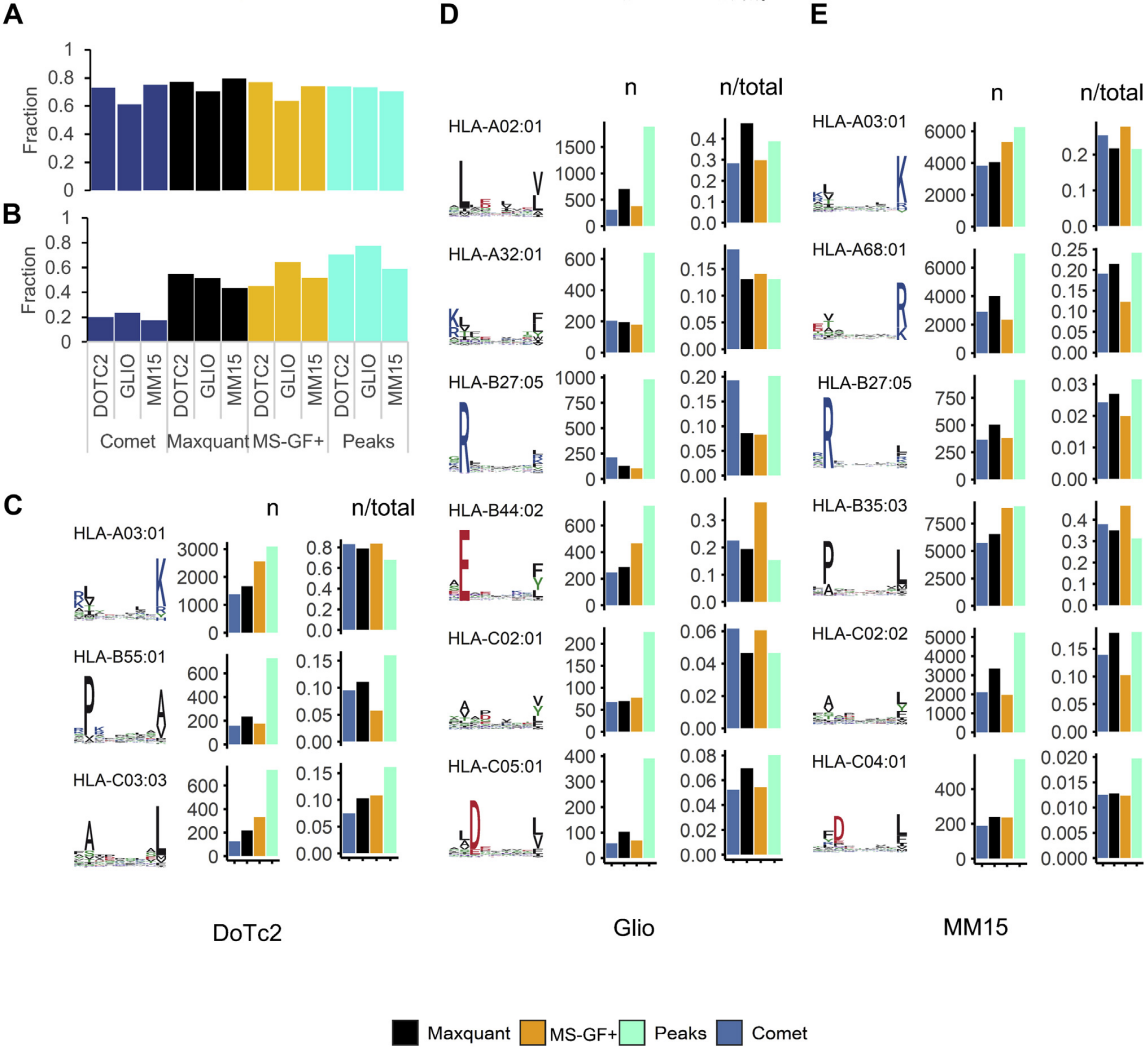
**Stratification of observed peptides by HLA allele using NetMHCpan binding prediction.***A*, proportion of all peptides predicted to bind (rank score < 0.5) to concomitant HLA molecules by NetMHCpan 4.1. *B*, proportion of predicted binders exclusively identified by each search engine. *C*–*E*, panels show from *left* to *right* the sequence logo for peptide 9-mer motif, total number (n), and proportion (n/total) of peptide sequences predicted to bind (rank < 0.5) to concomitant HLA molecules by NetMHCpan 4.1 for each immunopeptidomic dataset investigated.

Next, we used NetMHCpan 4.1 to deconvolute the HLA binding affinity of all 8 to 14 mer peptides in the DoTc2, GLIO, and MM15 datasets from the melanoma cohort (27). Given a NetMHCpan normalized affinity rank score of <0.5, we observed that for each search engine a similar proportion (0.61–0.79) of peptide sequences was predicted to bind to an allele (Fig. 2*A*). For search-engine-specific peptides, Peaks achieved a higher fraction of predicted binders when compared with COMET, MS-GF+, or Maxquant (Peaks = 58%–77%) (Fig. 2*B*). After stratifying peptides by the predicted HLA allele of origin, we found that the relative proportion of peptides assigned to each allele was varying for each search engine (Fig. 2, *C*–*E*). For example, COMET, Maxquant, and MS-GF+ identified a higher proportion of peptides that were predicted to bind A∗03:01 (Dotc2), A∗68:01 (MM15), and B∗44:02 (GLIO) than Peaks. In parallel, COMET, Maxquant, and MS-GF+ identified a lower proportion of B∗55:01, C∗03:03 (Dotc2), B∗27:05, C∗04:01 (MM15), B∗27:05, and C∗05:01 (GLIO) peptides when compared with Peaks (Fig. 2, *C*–*E*). Additionally, MS-GF+ identified a higher proportion of B∗44:02 peptides than in Peaks, Maxquant, or Comet. In order to validate that the observed differences were not introduced by biases in binding prediction, we performed this analysis with different NetMHCpan binding rank thresholds and found identical trends for all rank score cutoffs chosen (Fig. S5). We also cross-validated the NetMHCpan results with an unsupervised clustering approach, which assigns peptides to a sequence cluster independent of MHC binding prediction (MixMHCp). We observed a similar proportion of peptides from each search engine assigned to a recognizable motif by MixMHCp than we had obtained for NetMHCpan analysis and found overall correlation between both analyses in all three datasets (Figs. S6–S8).

**Fig. 3:**
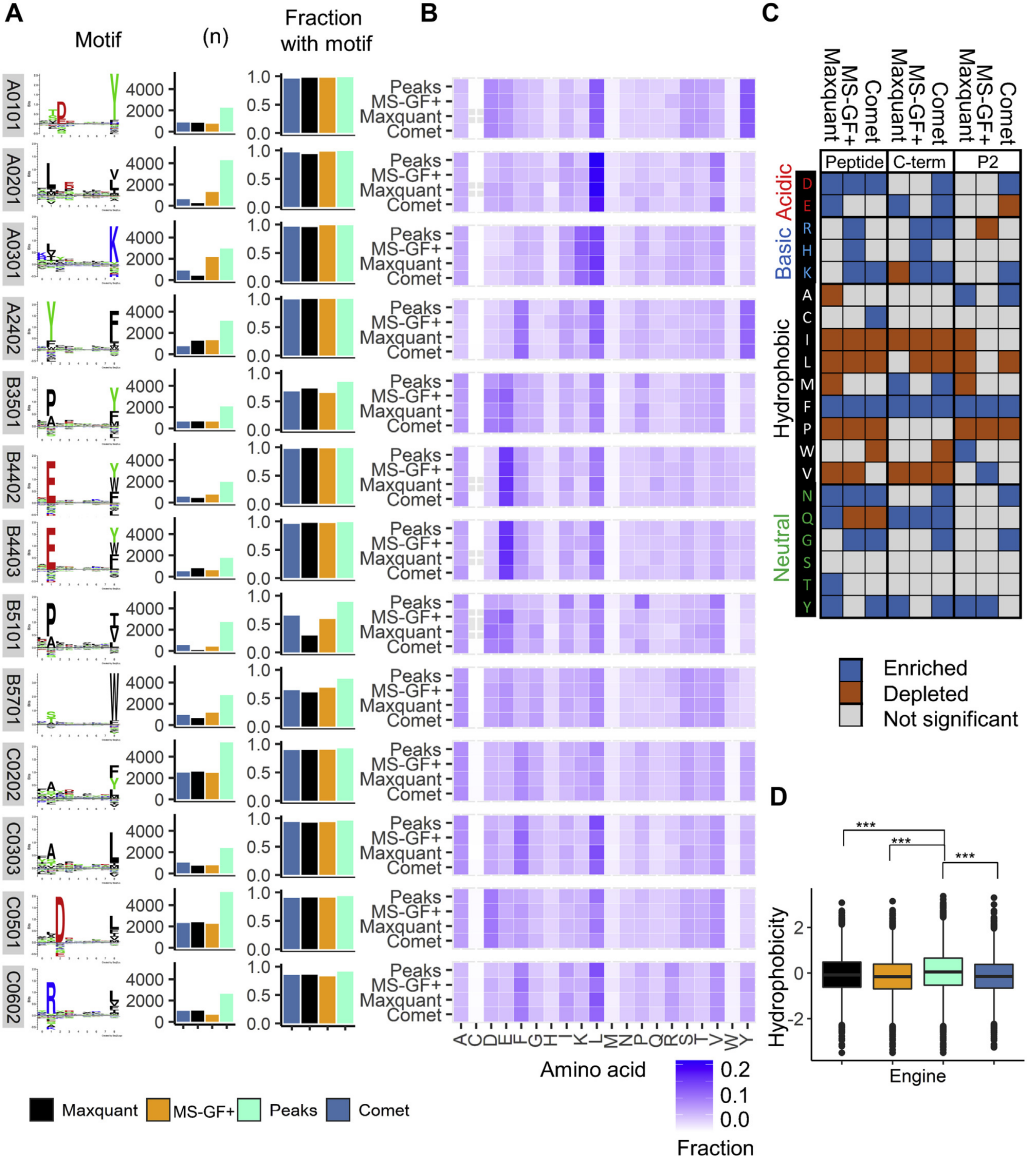
**Comparison of search engine results in single allele expressing cell line (SAEC).***A*, sequence motifs for peptides identified from data acquired on 13 different SAEC’s. *B*, bar plots showing the total number of peptides identified (*left*) and bar plots giving the proportion of 8- to 14-mer peptides that cluster into the single motif as determined by MixMHCp (*right*). *B*, heatmap showing the proportional frequency at which an amino acid is present in identified peptides, stratified by choice of search engine. *C*, composition profiler analysis results shown as a heatmap of amino acids that are enriched, depleted, or unchanged (*p* < 0.05, Bonferroni corrected). Peptides identified by Peaks were utilized as a background from which to compare the other three search engines. The entire peptide, C-terminal, and P2 amino acids were analyzed and plotted with search engine on the x-axis. *D*, boxplot of the GRAVY hydrophobicity score of peptides identified by each search engine (ANOVA with a Tukey's range test for multiple comparisons ∗Q < 0.05, ∗∗Q < 0.01, ∗∗∗Q < 0.001)

To explore these findings independent of HLA binding prediction and sequence clustering, we investigated immunopeptidomic data for 13 single allele expressing cell lines (SACL) with divergent peptide binding motifs PXD009531 and MSV000080527 (28, 29) (Fig. 3*A*). Consistent with the observations in “mixed” immunopeptidomes, Peaks achieved a higher number of peptide identifications for all datasets investigated, while MixMHCp analysis found that a similar and generally high proportion of these peptides contained the appropriate motif regardless of search engine choice (Fig. 3*A*). It stood out that all search engines identified the least peptides matching the relevant sequence cluster for B∗35:01, B∗51:01, and B∗57:01 containing P and W at P2 and C-terminus, respectively. We created heat maps to monitor the relative frequency of which specific amino acids were reported by each search engine, and we noted that other amino acids were also over- or underrepresented across the datasets (Fig. 3*B*). In order to assess statistically significant (*p* ≤ 0.001) enrichment and depletion of amino acids between the search engines, the Composition Profiler tool (18) was utilized. When comparing the peptide lists identified by Peaks against the three other search engines, we observed an overall reduced frequency of basic and acidic amino acid residues and an enrichment of hydrophobic residues (with exception of F, which was depleted) in the Peaks peptide lists. Specifically, a consistently higher frequency of L/I/P/V was reported in Peaks *versus* the three other search engines (Fig. 3*C*). Finally, we calculated the overall hydrophobicity (GRAVY) index for peptides and found that peptide identified by Peaks exhibited significantly higher hydrophobicity than those identified by MS-GF+, Maxquant, and Comet in alignment to the amino acid frequency analysis (Fig. 3*C*).

### Assessment of Search Engine Sensitivity and Validation of Observed Biases in Reported Allele-Specific Repertoires Using a Synthetic Standard Library for Four Common HLA Alleles

**Fig. 4:**
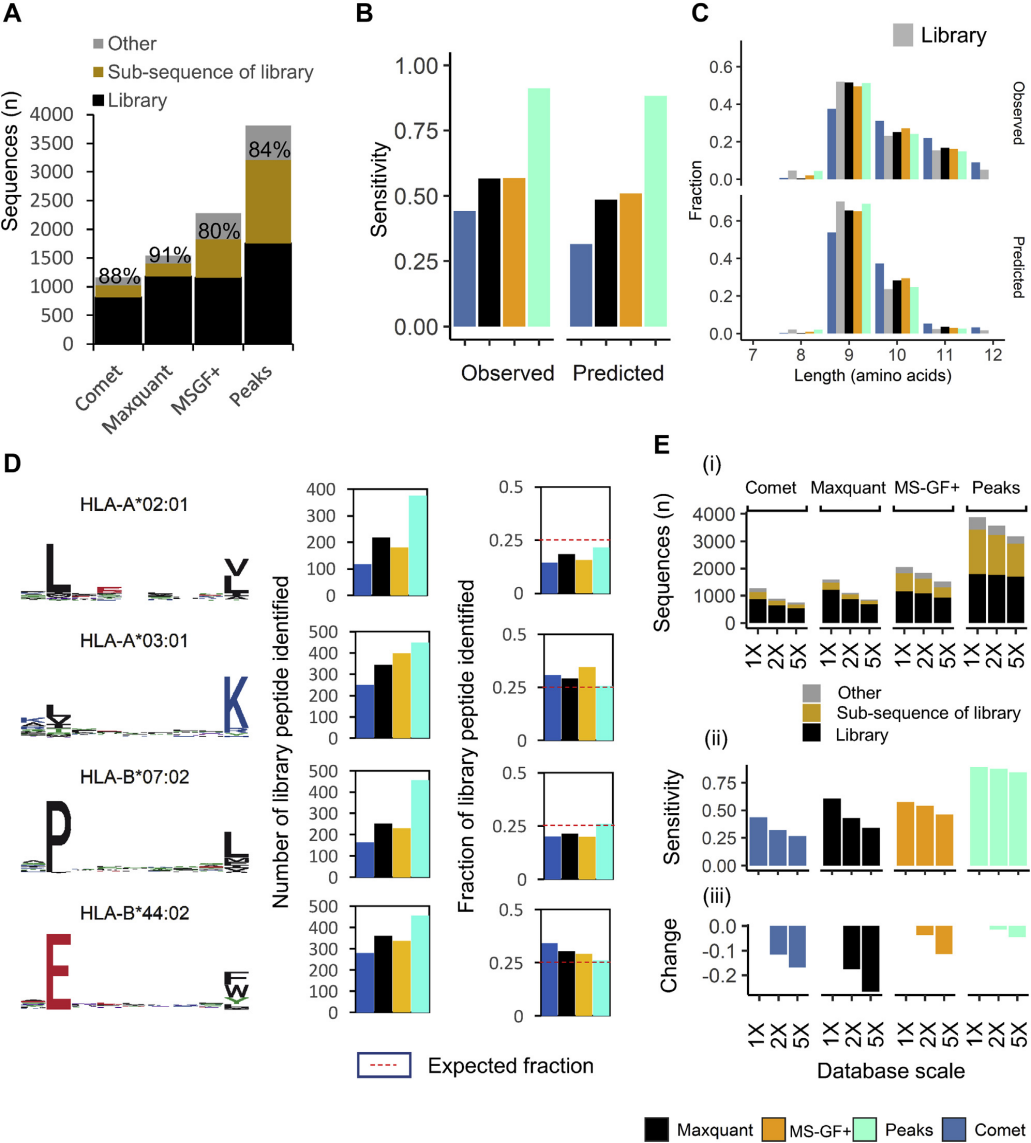
**Search engine sensitivity assessment using a synthetic standard library.***A*, total number of library target sequences (*black*), target subsequences (*gold*), and other sequences (*gr*e*y*) identified at 1% FDR. The % given is the total proportion that was either a target of synthesis or a sub-sequence of a target. *B*, fraction of library peptides identified by each search engine at 1% FDR cutoff, stratified by peptide origin for either “observed” in IEDB or “predicted” by NetMHCpan 4.1, as indicated. *C*, peptide length distribution identified by each search engine for each data set at 1% FDR cutoff, stratified by peptide origin for “observed” in IEDB or “predicted” by NetMHCpan 4.1, as indicated. *D*, the sequence logo for peptide 9-mer motif (left) and the number and fraction of target library peptides identified by each search engine at 1% FDR cutoff stratified by allele and peptide origin for “observed” in IEDB or “predicted” by NetMHCpan 4.1. The expected proportion of 0.25 is marked by a *red* dashed line. *E*, bar plot showing how database size affects (i) the number of peptides identified in the synthetic standard (library target sequences (*black*), subsequence (*gold*), and other peptides (*gr*e*y*) identified at 1% FDR, (ii) the fraction of true-positive (sensitivity) library peptides identified, (iii) the relative change in sensitivity.

To validate the observed biases between the different search engines, we synthesized a library consisting of 2000 peptides for four frequent and diverse HLA molecules with distinct anchor residues (A∗02:01, A∗03:01, B∗44:02, B∗07:02). We decided to partition the library in two main peptide pools: Using IEDB we chose at random 1000 peptides previously observed in mass spectrometry experiments (250 for each selected allele, “observed” partition), and 1000 peptides that had not been previously observed that originated from the same source proteins but were predicted to bind to the same HLA allele (250 for each selected allele, “predicted” partition) (Fig. S9*A*). This workflow resulted in a library that exhibited characteristic of HLA-associated peptides in length and anchor residues for the chosen alleles (Table S1 and Fig. S9*B*). The length distribution of peptides measured previously (observed) had a higher relative proportion of 10 to 14 mers when compared with peptides predicted by NetMHCpan (predicted) (Fig. S9*B*). After LC-MS acquisition (see Experimental Procedures for details) and identical data processing, we observed substantially more hits for library peptides with Peaks, which was similar to our observations in mixed immunopeptidomic datasets (Fig. 4*A*). With these data we determined the proportion of true-positive peptide identifications made by each engine at <1% FDR (COMET = 41%, Maxquant = 59% MS-GF+ = 58% and Peaks = 68%) and observed the highest identification rate for Peaks (Fig. 4*B*). This observation was consistent for both “predicted” and “observed” partitions of the library (Fig. 4*B*) and, as expected, improved sensitivity and more accurately reflected the length distribution of the synthetic peptide library (Fig. 4*C*).

All search engines identified a considerable number of additional peptide sequences that were not targeted for synthesis during library creation (Fig. 4*A*). This indicated that either the library contained many other peptides next to the anticipated synthesis targets (hereinafter termed “target” peptides) or that false-positive peptide sequences were reported despite the application of a global FDR of 1%. Sequence analysis of the additional peptide identifications revealed that a high proportion of these sequences were subsequences of the target peptide sequences and that they were overall shorter and lower in abundance (Fig. 4*A*, Figs. S10, *A*–*C* and S11). If we combined all sequences that were likely physically present in the library (target peptides and any subsequences of such) 80% to 91% of all peptides identified by a search engine could be accounted for (Fig. 4*A*). After stratification by allele, we observed that Peaks more accurately reflected the expected equal proportion of peptides binding to each allele as present in the library (Fig. 4*D*). All search engines underestimated the proportion of the hydrophobic A∗02:01 peptides (which has mainly L at P2, and L/V at the C-terminal anchor), and Peaks identified the highest proportion of A∗02:01 peptides. Maxquant, Comet, and MS-GF+ also underestimated hydrophobic B∗07:02 peptides, that binds predominantly peptides with a P at P2; and overestimated the proportion of basic/acid A∗03:01, B∗44:02 peptides (Fig. 4*D*). On further examination we also found that peptides from the four different alleles resulted in different score distributions for the same search engine, with synthetic peptides that have hydrophobic anchors scoring worse than their polar counterparts (Fig. S12). These observations reflected the search engine bias observed for peptides with regarding amino acid anchors in both the mixed and single allele immunopeptidomic datasets.

Identification of neoantigens in immunopeptidomic data often requires the interrogation of larger search spaces generated from bespoke genomic analyses. Since we had previously observed a possible dependency of the search engine performance on the search space in tryptic *versus* nontryptic analyses, we assessed the effect of a larger search space on peptide identification sensitivity. We used the original human SwissProt database and expanded it by randomization to contain two and fivefold the number of unique protein sequences and amino acid residues. We then used these significantly larger databases, both still containing the original SwissProt defined human proteome and additional randomized sequences, and appended an equally sized, fully randomized database for unbiased FDR evaluation as before. We reanalyzed the data acquired for the synthetic peptide library with each search engine. Results demonstrate that increasing search space reduced the overall number of peptide identification reported for each search engine (Comet 30 and 41%, Maxquant 30 and 44%, MS-GF+ 12 and 17%, Peaks 8 and 18% for 2 and 5x databases, respectively) (Fig. 4*E*). This affect was also reflected in a reduction in sensitivity (identification rate of target peptides): Comet 12 and 17%, Maxquant 18 and 27%, MS-GF+ 4 and 11%, Peaks 2 and 5% for the 2 and 5x database expansion, respectively. Overall Peaks exhibited the lowest loss of sensitivity in the larger search spaces, and the effects of DB size appeared to be independent of allele (Fig. 4*E*).

## Discussion

Ideally, a database search analysis tool should provide a sensitive and representative identification of as many correct peptide spectrum matches as possible (30). In the immunopeptidomic search space, the four programs investigated here varied considerably in sensitivity and the proportion of peptides assigned to each HLA allele, while performing equally well for the identification of tryptic peptides. Using single allele expressing cell lines, we recapitulated differences in sensitivity and allele-specific differences observed in mixed allele datasets. We further observed that the bias in identification of allele-specific peptide fractions is related to the biochemical properties of amino acids in peptides and is driven by an underrepresentation of hydrophobic amino acids. We confirmed our findings through analysis of a synthetic peptide library.

Using the widely accepted target/decoy approach to control FDR, a comparison of four programs investigated here identified considerable variation in sensitivity and the proportion of peptides assigned to each HLA allele in immunopeptidomics datasets. Higher sensitivity was consistently achieved by analysis with Peaks, supporting previous observations (7). We implemented two alternative allele deconvolution algorithms (NetMHCpan and MixMHCp) and demonstrated that most of the additionally identified peptides by Peaks were highly likely to bind to HLA alleles present in the associated samples, indicating an overall high accuracy in sequence assignment as also observed by others (7). Additional peptides identified by Peaks often had lower scores, indicating that Peaks can stratify true from false peptide spectrum matches more accurately despite poorer spectrum quality. In further support of this, increasing the search space or a lowering the signal intensity had a much lower effect on sensitivity in Peaks compared with other search engines. This indicates that the Peaks’ peptide scoring algorithm can maintain sensitivity in large search spaces or where spectrum quality is lower. This hypothesis was supported through our analysis of scan fate at variable FDR cutoffs, in which a generally high proportion of peptides identified by Peaks at FDR <1% were also matched by the other search engines at a relaxed FDR of 5% (Fig. S4). These observations support the idea that implementation of “database independent score(s)” in a peptide identification algorithm can greatly improve the sensitivity of large meta-immunopeptidomic studies (31).

Improvements in the sensitivity of peptide identification by rescoring through combining search engines have been observed previously (13). Additionally, rescoring based on semisupervised machine learning, where algorithms are trained to discriminate between correct and decoy spectrum identifications, has been developed for proteomic (32, 33), and immunopeptidomic datasets (6, 8). Recently, retention time and the Percolator rescoring information was applied to search results from Comet, leading to increased sensitivity for immunopeptidomic data to a similar performance than Peaks (7). Importantly, a deep learning approach integrating nontryptic peptide fragmentation data led to highly improved identification rates in immunopeptidomics datasets (14).

Beyond sensitivity, the extent of peptides reported by the tested search engines to each of the alleles varied. The number of naturally presented peptides by each HLA allele present in the sample is driven by differences in HLA allele expression levels and the peptide copy number. It is likely that lower abundant and poorly detected peptide species are less efficiently identified and that these groups will benefit most from Peaks analysis or analysis utilizing novel algorithms that are able to address these specific challenges in immunopeptidomics datasets (7, 13). Here, our observation contrasted with data reported by Bichmann *et al.*, (7) where no allele bias was reported. We observed that search engines identified peptides with differing sensitivity that depended on amino acid composition, with some algorithms preferring peptides containing charged amino acids over hydrophobic residues. The underlying mechanism for this bias is currently unknown but could arise from probabilistic models based on amino acid frequency or assumptions about peptide fragmentation used to train/develop the search engines tested (22, 34, 35). In practice, the presence of basic amino acid side chains enhances peptide ionization and fragmentation resulting in rich spectral quality, whereas hydrophobic residues are uncharged and may influence charge and proton mobility, generally resulting in less informative spectra (36, 37). The influence that amino acids have on fragmentation is well reviewed (38), and given our observations, it is highly likely that with current tools the genetic makeup of *HLA* loci is directly affecting the effort to sequence and accurately report the immunopeptidome. Toward resolving these effects, work done by Bichmann *et al.*, (7) shows that rescoring peptides initially identified by Comet through post-hoc training not only results in a sensitivity equivalent to that observed in Peaks, but appears to result in a similar proportion of peptides assigned to each allele. Additionally, recent efforts to use spectral libraries of synthetic peptides to train prediction algorithms for peptide fragmentation provided greater sensitivity for proteomics and immunopeptidomics datasets and will have a high impact on the field of immunopeptidomics (14, 39, 40).

Since the evaluated search engines were updated while this manuscript was under review, we have compared the performance of the latest release of each search engine (as of June 1, 2021) with the versions used for data analysis in this manuscript. We observed that both sensitivity and peptide repertoire bias for each engine were almost identical to that we observed in the older versions and reported in this manuscript, outlining that the biases have so far not been addressed (Fig. S13).

In summary, our study highlights limitations of proteomic search engines for the analysis of immunopeptidomics datasets. There is an urgent need for the development of novel or adapted search engines that can provide high sensitivity and reproducibility for analysis of the large and diverse immunopeptidomic space where distinct variations in amino acid composition often occur and hamper their unbiased identification by classical approaches.

## Data Availability

The mass spectrometry proteomics data have been deposited to the ProteomeXchange Consortium (http://proteomecentral.proteomexchange.org) via the PRIDE partner repository with the dataset identifier PXD025655.

## Supplemental data

This article contains supplemental data.

## Conflict of interest

N. T. is directing immunopeptidomics research at Enara Bio part-time and serves on the Scientific Advisory Boards of Enara Bio and T-Cypher Bio. N. T. is consultant to Hoffman-La Roche and Grey Wolf Therapeutics. All other authors declare no conflict of interest.
