# Supplementary material for: The Choice of Search Engine Affects Sequencing Depth and HLA Class I Allele-Specific Peptide Repertoires
Source: Mol Cell Proteomics. 2021 Jul 23;20:100124. doi: 10.1016/j.mcpro.2021.100124 (PMC8724928; doi:10.1016/j.mcpro.2021.100124)
Supplement: Supplemental Figures S1–S13 [file mmc1.pdf]

Supplemental data

The Choice of Search Engine Affects Sequencing Depth and HLA Class I Allele-Specific Peptide Repertoires

R Parker[1*], A Tailor[1], X Peng[1], A Nicastri[1], J Zerweck[2], U Reimer[2], H Wenschuh[2], K Schnatbaum[2] and N Ternette[*1]

Figures S1-13

Tables S1 (See Table S1.xlsx)

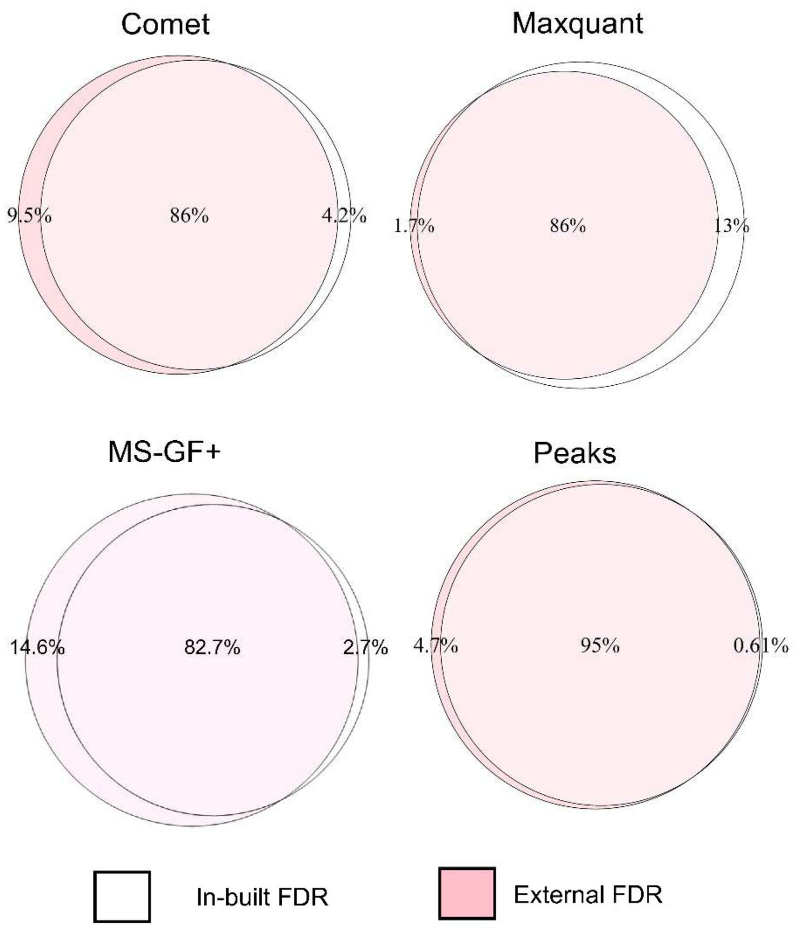

Figure S1: Consensus between in-built software calculated FDR strategies. Venn-diagram showing overlap between peptide sequences identified by our universally applied external FDR calculations and software specific in-built FDR for DoTc2 cell line immunopeptidome data.

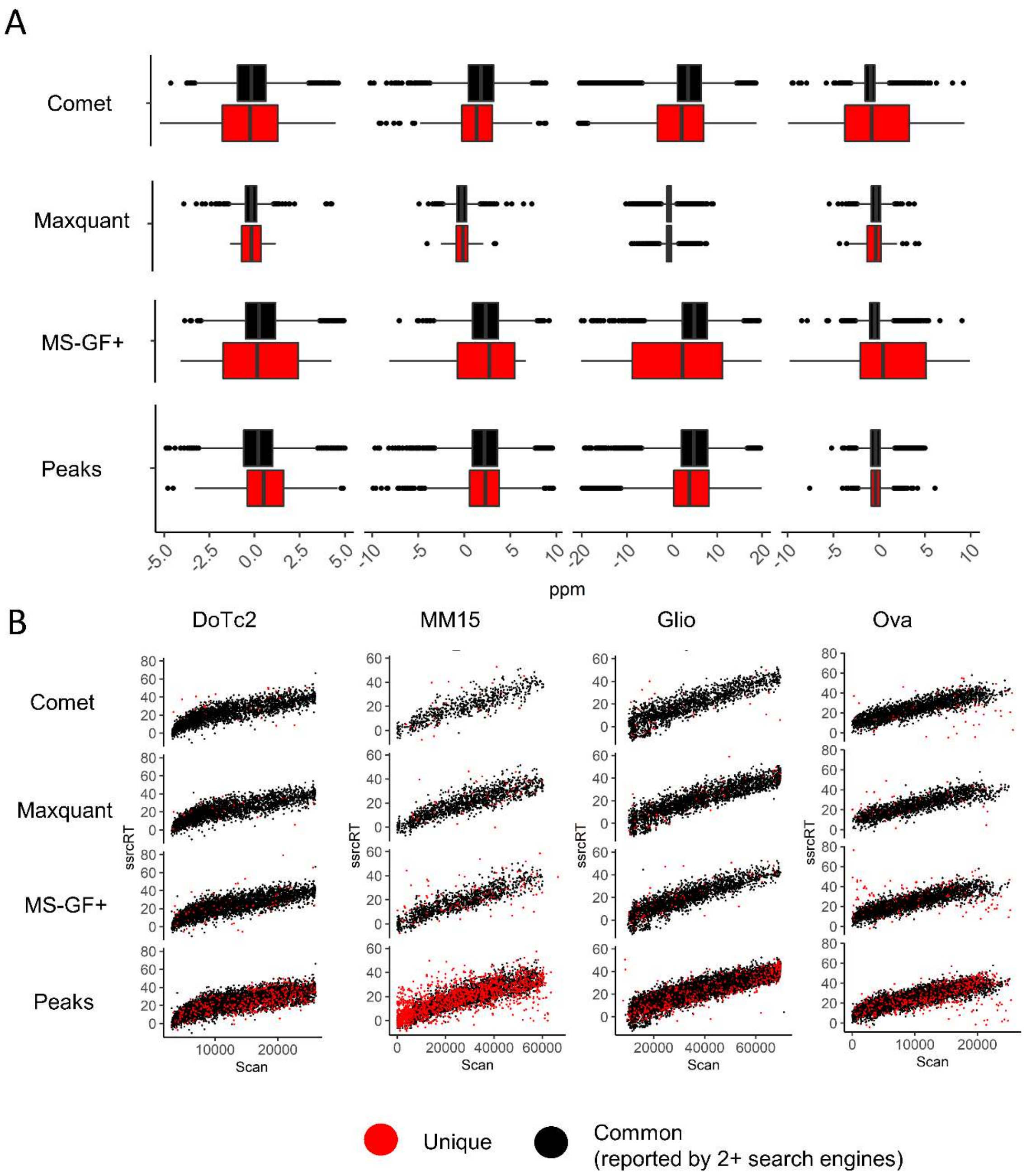

Figure S2. Mass errors and retention time prediction reported for common and unique peptide identifications for each search engine. (A) Boxplot of mass accuracy (ppm) for peptides identified by each search engine in indicated datasets. Peptides are stratified by (common, black) those identified by more than one search engines or (unique, red) those identified only by the search engine indicated. (B) Scatter plot of predicted peptide HPLC retention time (y=ssrcRT) with experimental HPLC retention (x=scan) for peptides identified by each search engine in the indicated datasets. Peptides are stratified by (common, black) those identified by all search engines or (unique, red) those identified only by the search engine indicated.

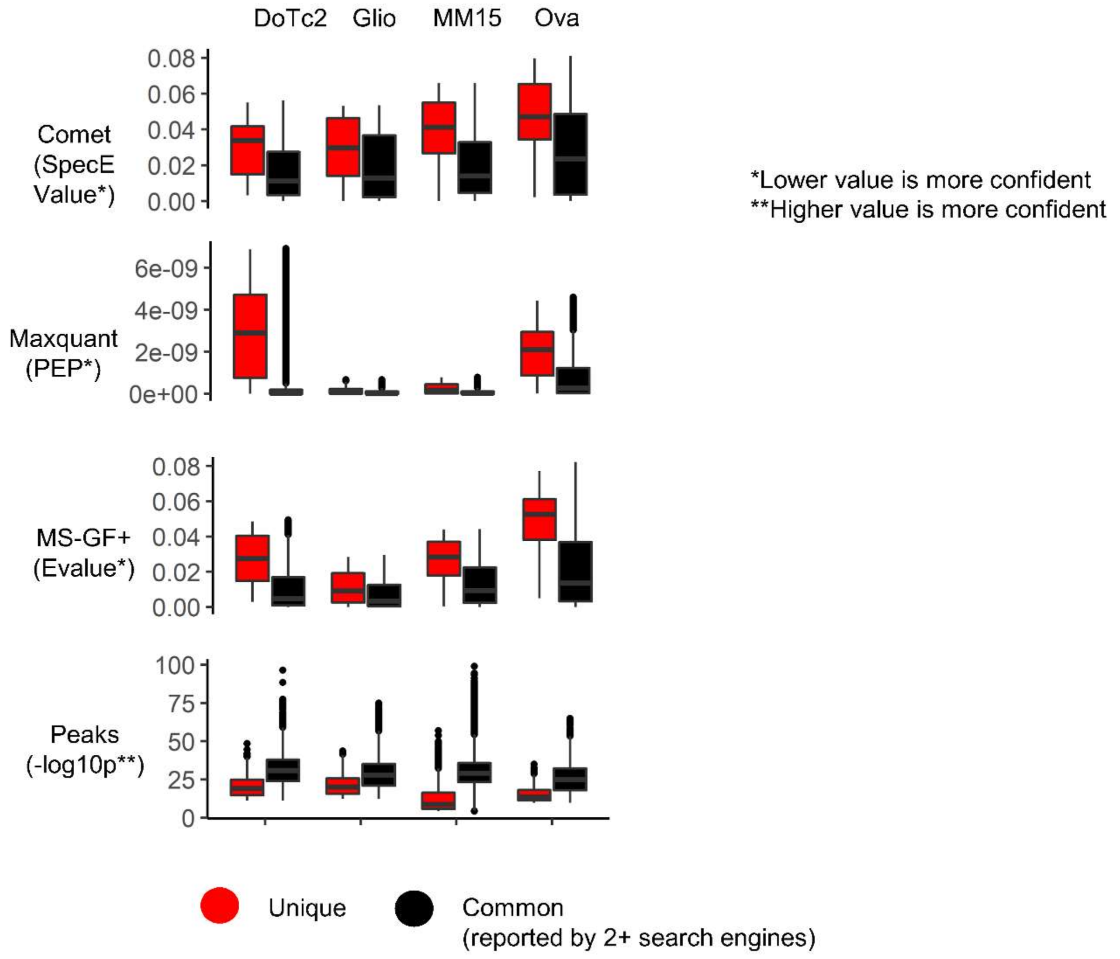

Figure S3. Score reported for common and unique peptide identifications: (A) Boxplot of search engine score for peptides identified by each search engine in four immunopeptidomic datasets. Peptides are stratified by (common, black) those identified by more than one search engine or (unique, red) those identified only by the search engine indicated.

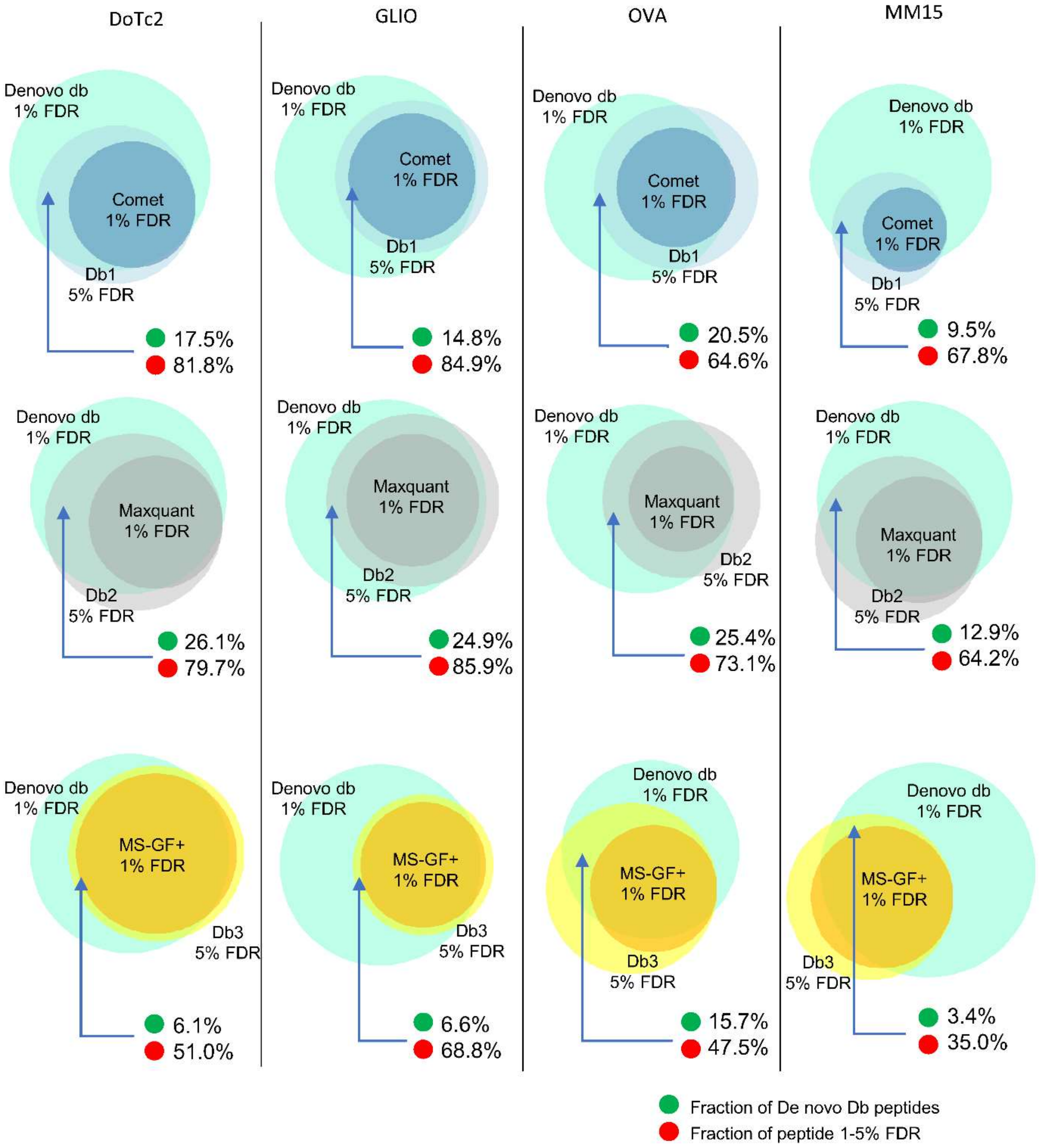

Figure S4. PSM fate analysis. Proportional Venn diagrams showing the intersection of peptide spectrum matches (PSMs) determined by each search engine and immunopeptidomic dataset after filtering at <1% in Peaks, <1 and <5% FDR in Maxquant, MS-GF+ and Comet. Green is the fraction of total Peaks peptides recovered; red is the fraction of peptides identified at 1-5% FDR peptides also found by Peaks.

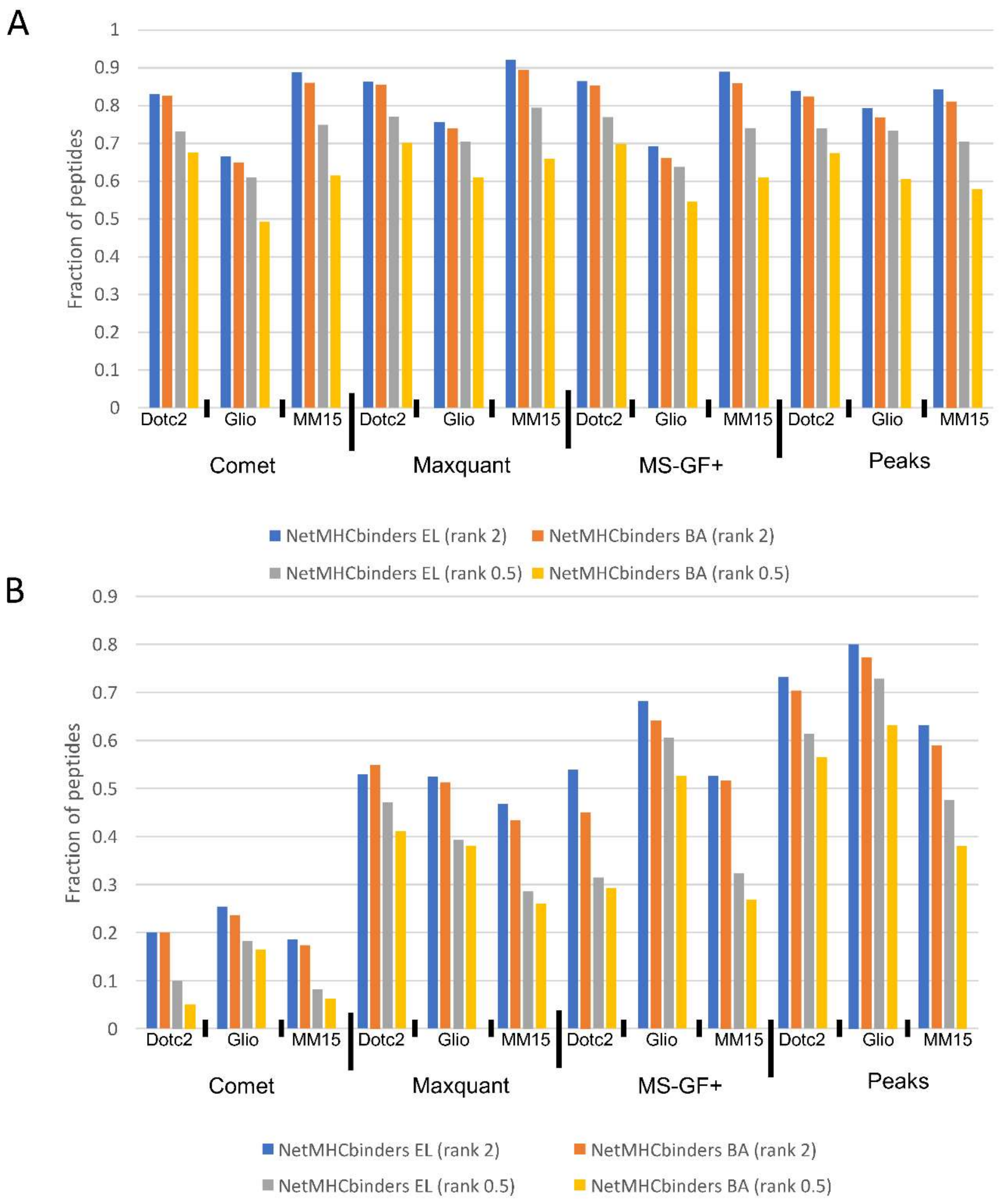

Figure S5. Comprehensive NetMHCpan binding prediction results: Peptide's length 7-14 mer were analysed using NetMHCpan 4.1 given the alleles known to be present within each sample. (A) Shows the fraction of all peptides (B) shows peptides unique to a search engine, that are predicted to bind to an allele utilising the eluted ligand (Low affinity EL rank <2 Blue, High affinity EL rank <0.5 Gray) or Binding affinity (Low affinity BA rank < 2 Orange, High affinity BA rank < 2 Gold) to indicate affinity to concomitant HLA molecules.

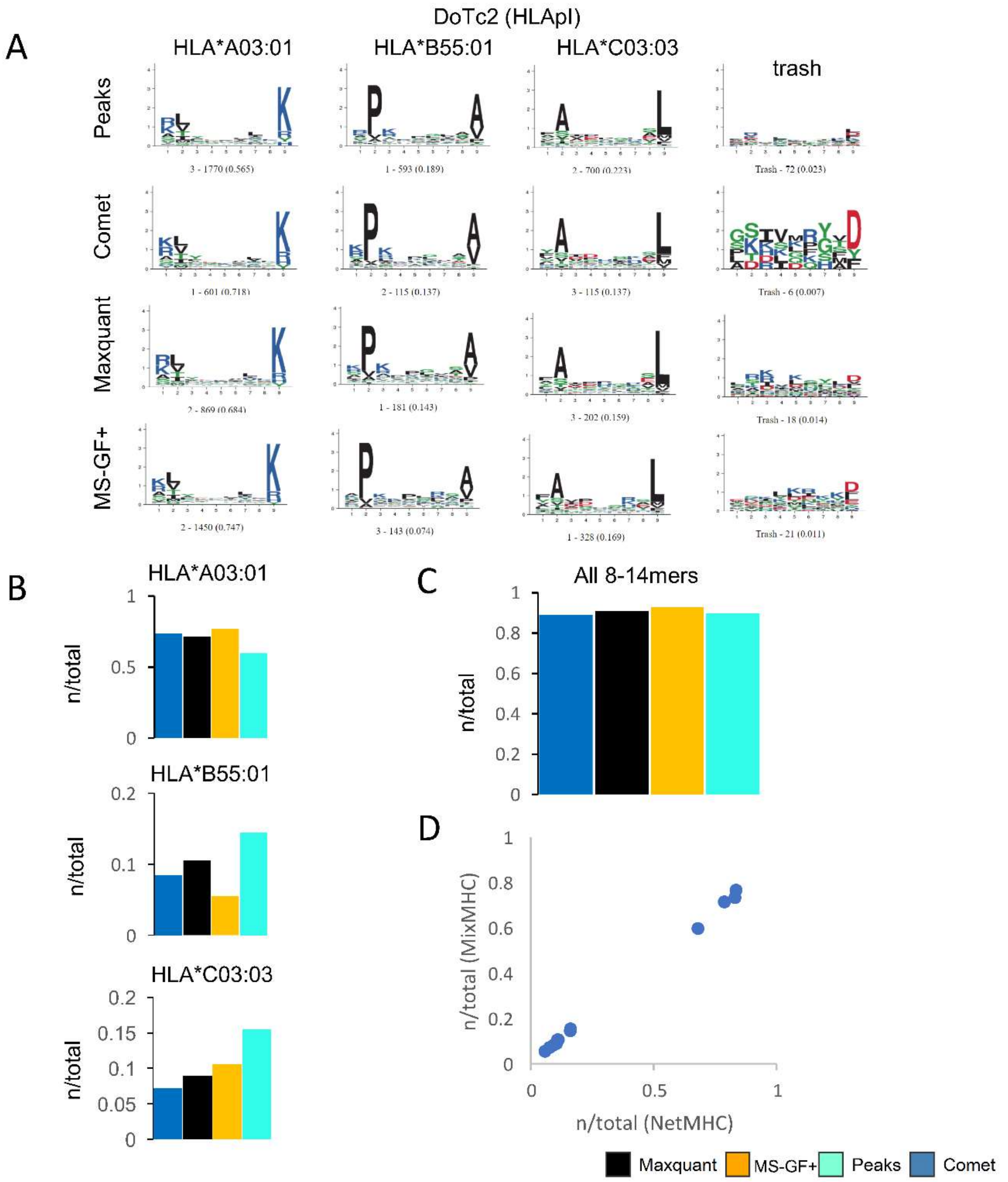

Figure S6. MixMHCp analysis of DoTc2 immunopeptidomic dataset after analysis by different search engines. (A) Sequence motifs generated by MixMHCp for all 7-14-mer peptides (9-mers shown) identified by each search engine, motifs were manually annotated as belonging to one of the known concomitant HLA molecules in the sample, a Trash cluster with no motif was assigned. (B) Fraction of peptides assigned to each allele for each search engine result. (C) Fraction of the total number of peptides not in the trash cluster. (D) Scatter plot of the fraction of peptides assigned to each allele for each search engine result when analysed by either NetMHCIpan (x-axis) or MixMHCp (y-axis).

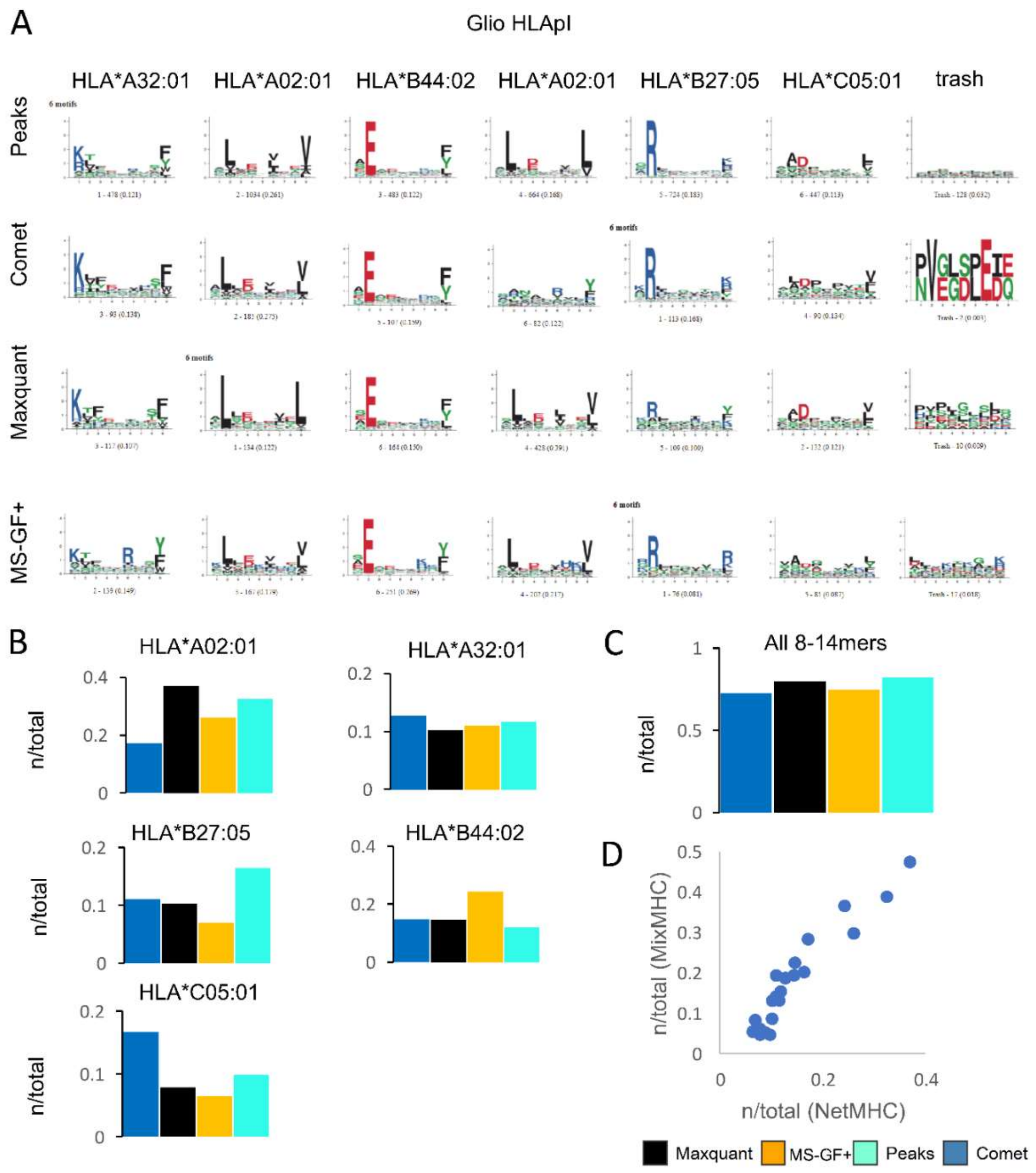

Figure S7. MixMHCp analysis of Glioblasma (Glio) immunopeptidomic dataset after analysis by different search engines. (A) Sequence motifs generated by MixMHCp for all 7-14-mer peptides (9-mers shown) identified by each search engine, motifs were manually annotated as belonging to one of the known concomitant HLA molecules in the sample, a Trash cluster with no motif was assigned. (B) Fraction of peptides assigned to each allele for each search engine result. (C) Fraction of the total number of peptides not in the trash cluster. (D) Scatter plot of the fraction of peptides assigned to each allele for each search engine result when analysed by either NetMHCIpan (x-axis) or MixMHCp (y-axis).

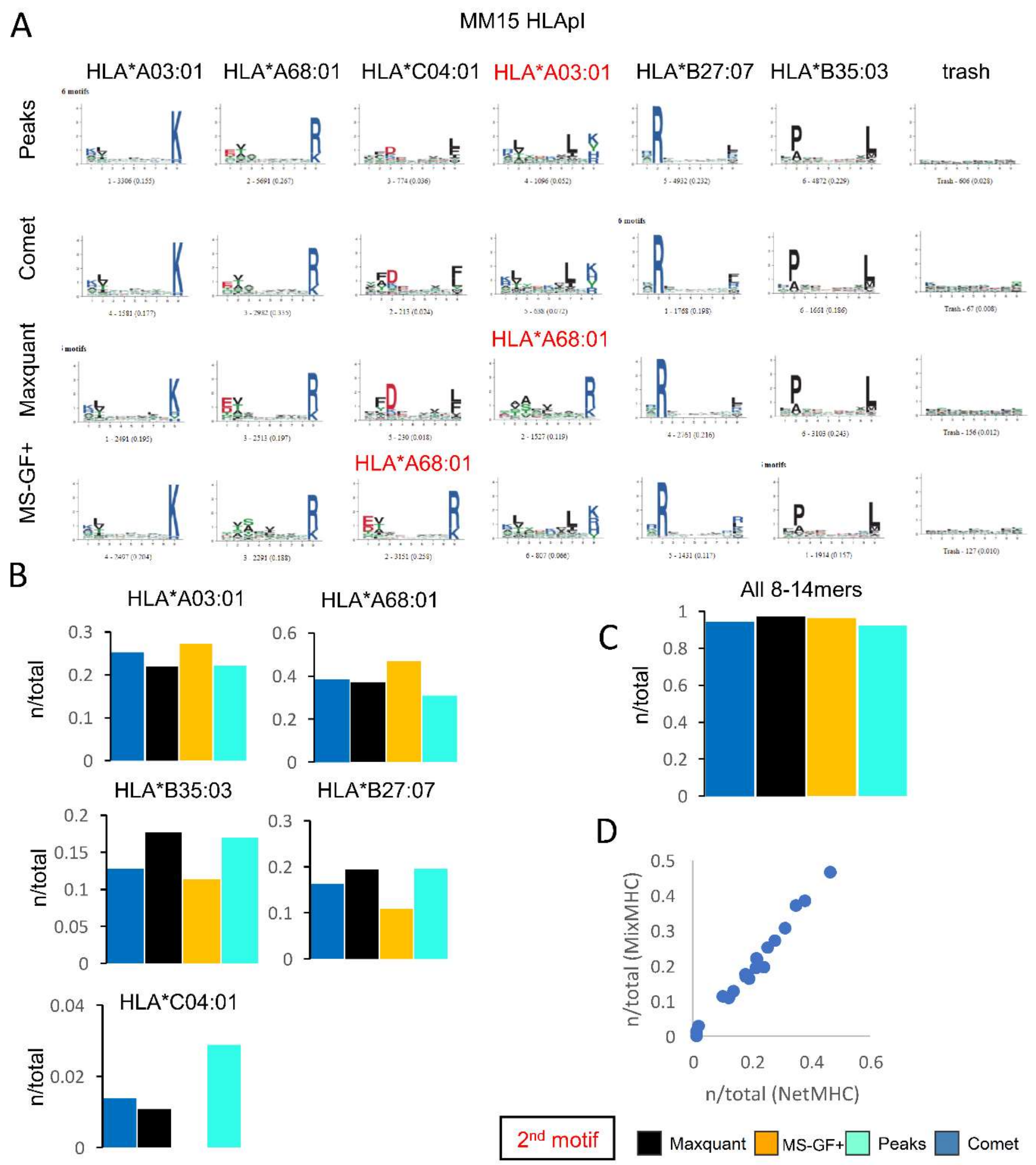

Figure S8. MixMHCp analysis of Melanoma (MM15) immunopeptidomic dataset after analysis by different search engines. (A) Sequence motifs generated by MixMHCp for all 7-14-mer peptides (9-mers shown) identified by each search engine, motifs were manually annotated as belonging to one of the known concomitant HLA molecules in the sample, a Trash cluster with no motif was assigned. Where a 2nd motif is present for an allele it is indicated in red text. (B) Fraction of peptides assigned to each allele for each search engine result. (C) Fraction of the total number of peptides not in the trash cluster. (D) Scatter plot of the fraction of peptides assigned to each allele for each search engine result when analysed by either NetMHCIpan (x-axis) or MixMHCp (y-axis).

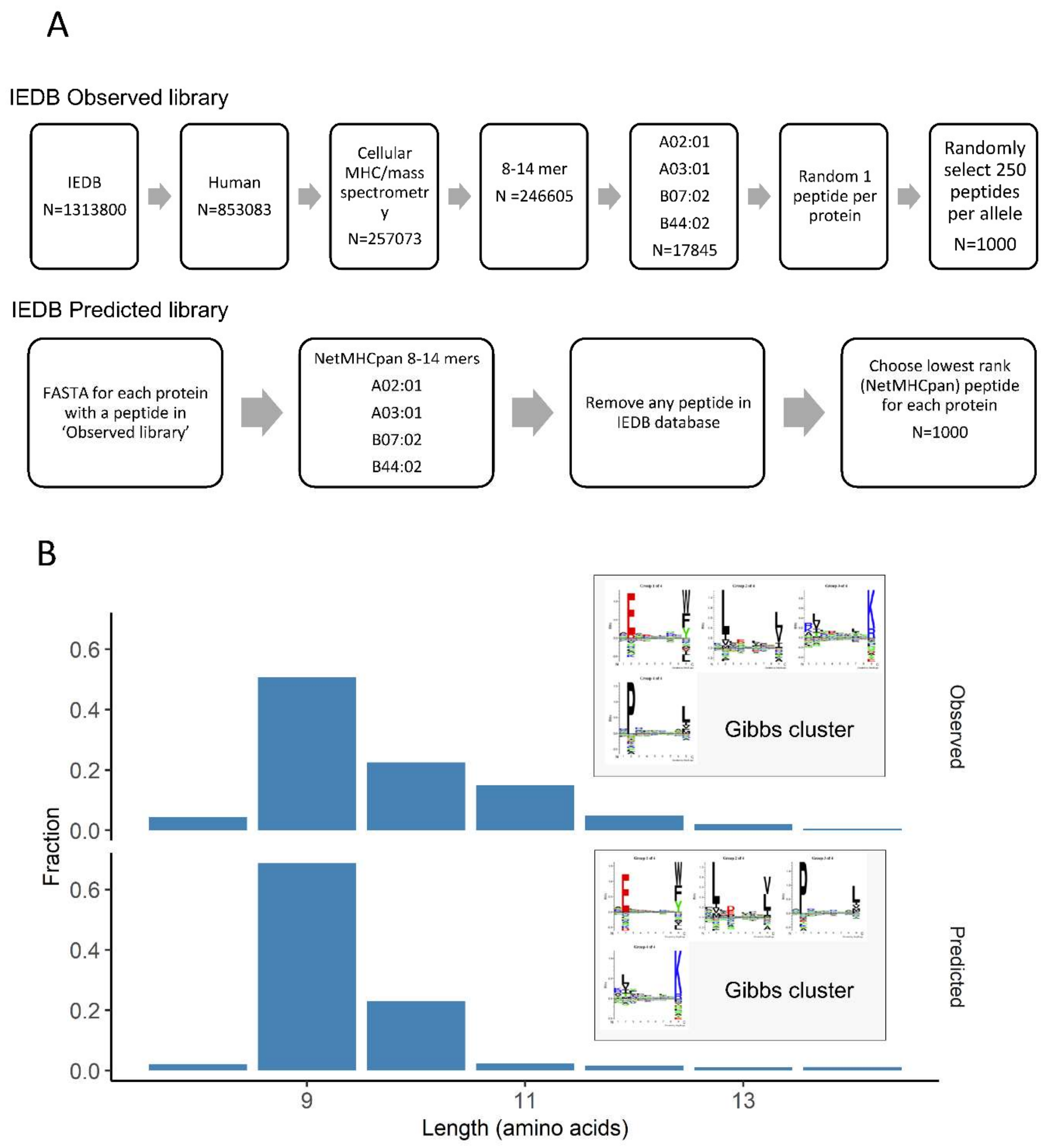

Figure S9. Design and overview of synthetic peptide standard. (A) Flow diagram depicting the key steps taken to choose peptides for creation of a 2000 synthetic peptide library of 1000 IEDB observed or 1000 predicted sequences. (B) Histogram showing the relative frequency of sequence length for peptides in the synthetic peptide library of 1000 IEDB observed or 1000 predicted sequences, the figure is inlayed with the sequence motifs detectable by Gibbs clustering within each library.

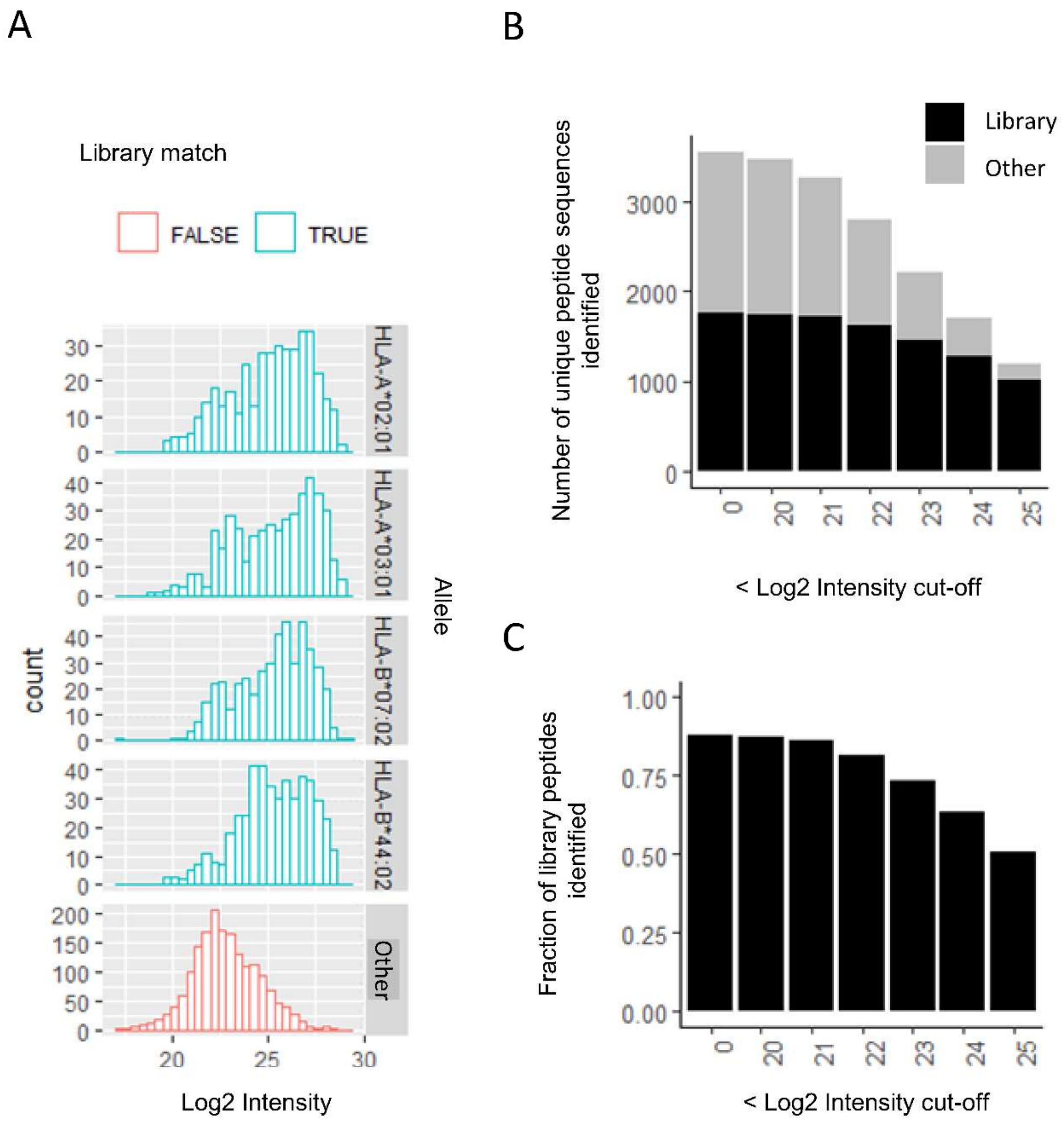

Figure S10. Quantitative analysis of peptide sequences identified in the synthetic peptide standard by Peaks. (A) Abundance distribution of peptide sequences that were targets of synthesis in the peptide library (TRUE, blue) stratified by HLA allele, and those that were not targets of synthesis (FALSE, red, Other). (B) Number of peptide sequences identified by Peaks that that were targets of synthesis in the peptide library (black) or were not targets of synthesis in the peptide library (other, grey), as a function of increasing signal intensity (C) Fraction of library peptides identified by Peaks over increasing signal intensity thresholds.

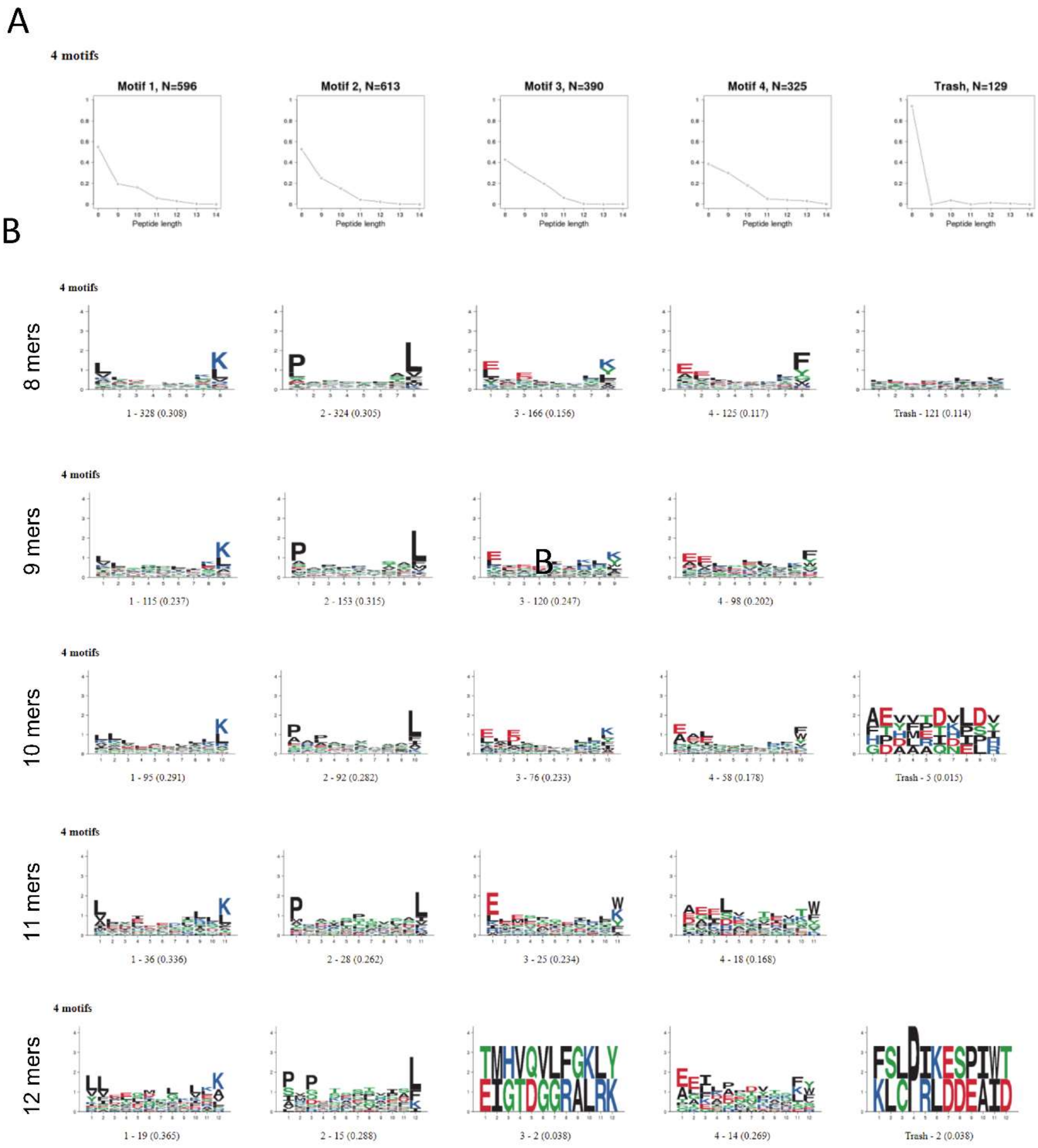

Web logo of none library 8-12 mers peptides identified at 1% FDR

Figure S11. MixMHCp analysis of immunopeptidomic standard additional peptides identified by LC-MS but not targeted for synthesis. (A) Histogram of length distribution for peptides found in 4 different motifs or the trash (no-motif) clusters. (B) Sequence motifs generated by MixMHCp for all 8-12-mer peptides identified by each search engine and a Trash cluster with no motif was assigned.

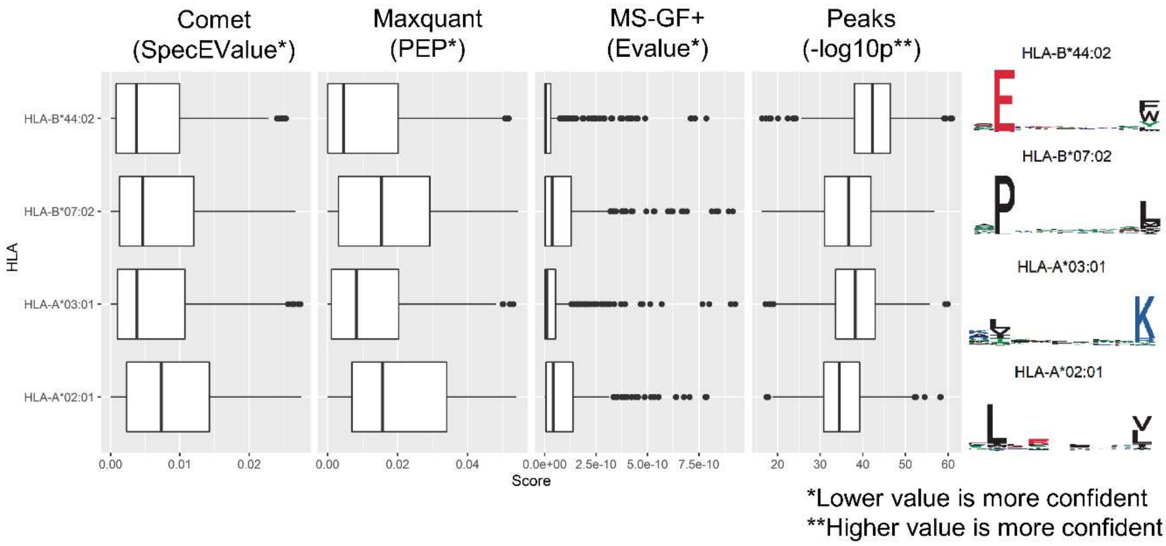

Figure S12. Stratification of search engine score by HLA. Boxplots of scores used to assign peptide confidence stratified by search engine (x-axis) and allele (y-axis**).**

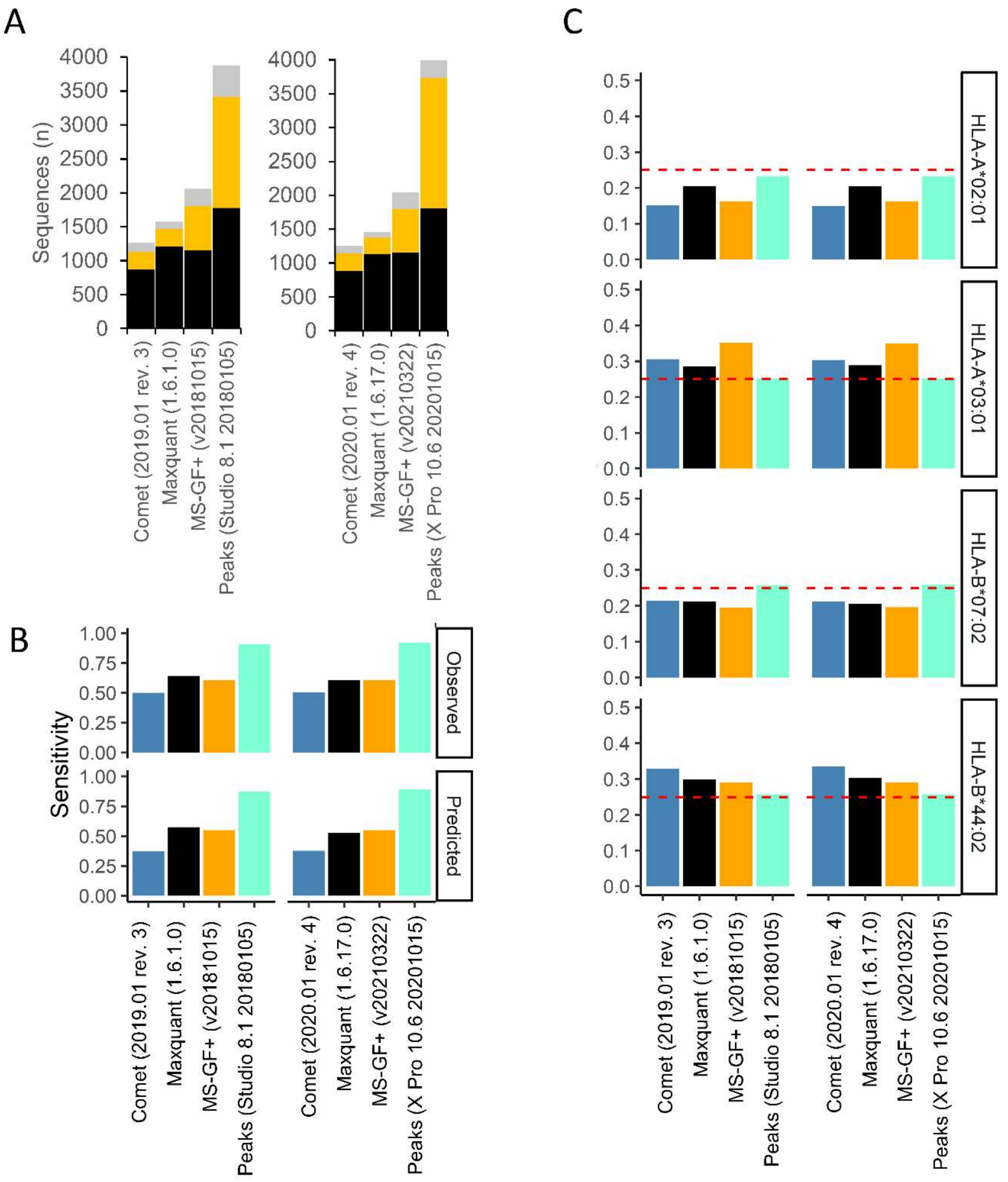

Figure S13. Search engine sensitivity assessment using a synthetic standard library for the latest release of each search engine. (A) Total number of library target sequences (black), target subsequences (gold), and other peptides (grey) identified at 1% FDR for each search engine version as indicated. (B) Fraction of library peptides identified by each search engine at 1% FDR, stratified by peptide origin for either "observed" in IEDB or "predicted" by NetMHCpan 4.1 and search engine version as indicated. (C) The fraction of target library peptides identified by each search engine at 1% FDR stratified by allele and peptide origin for "observed" in IEDB or "predicted" by NetMHCpan 4.1 and search engine version as indicated. The expected proportion of 0.25 is marked by a *red* dashed line.
